# Myocardial Iron overload quantification in a developing country: Tunisian first experience with financial challenges

**DOI:** 10.1186/1532-429X-17-S1-W21

**Published:** 2015-02-03

**Authors:** Ismahen Ben Yaacoub-Kzadri, Chawkat Ramadane, Aicha Ben Miled, Najla Mnif

**Affiliations:** 1 Radiology, Charles Nicolle Hospital, Tunis, Tunisia

## Background

Iron chelation treatment has increased life expectancy of patients with beta thalassemia major. Accurate assessement of heart iron overload is essential for managing chelation therapy. Nowadays, MRI-based iron overload quantification became the gold standard technique. However it remains too expensive in countries that cannot afford such technology, which ironically are endemic countries of thalassemia. The aim of our study is to evaluate the impact of measuring heart iron concentration by the means of MRI using a "low cost" technique in a developing country like ours with few financial resourses.

## Methods

We studied prospectively 101 patients with beta thalassemia major. Serum ferritin and left ventricular ejection fraction were compared to cardiac T2* and myocardial iron concentration (MIC) assessed with a validated technique based on MRI relaxometry by the means of single echo T2* sequences with crescent TE; and a free Excel speadsheet for post processing. Liver iron overload quantification was performed during the same examination and using the same method. The total duration of the examination was 20 to 25 min and post processing 5 to 10 min.

## Results

A significative negative correlation was found between serum ferritin levels and cardiac T2* and MIC values. Heart and liver T2* values showed a significative positive correlation. All patients with an altered LVEF had a heart T2*< 10ms. Patients with moderate and severe myocardial iron overload benefited from optimization of their chelation therapy. Close monitoring of severely overloaded patients was recommended.

## Conclusions

Our results suggests that the single echo T2* technique coupled with Excel post processing is a fast, non-invasive, reliable and low cost method for the assessment of heart iron concentrationn, appropriate to the budget of the developing countries.

## Funding

N/A.

